# 394. Consistency of AZD7442 (Cilgavimab/Tixagevimab) Pharmacokinetics Across Prophylaxis and Treatment and Adult and Pediatric Participants: Application of Population Pharmacokinetics to Enable Rapid Decision-Making During the COVID-19 Pandemic

**DOI:** 10.1093/ofid/ofad500.464

**Published:** 2023-11-27

**Authors:** Lindsay E Clegg, Oleg Stepanov, Henning Schmidt, Ventakesh Pilla Reddy, Weifeng Tang, Michael Gibbs, Sangeeta Sedani, Antonella Nadia Tuillio, Sam Sadow, Nuria Martinez-Alier, Taylor Cohen, Saul N Faust, Mark T Esser, Mats Någård

**Affiliations:** AstraZeneca, Gaithersburg, Maryland; AstraZeneca, Gaithersburg, Maryland; IntiQuan GmBH, Basel, Basel-Stadt, Switzerland; AstraZeneca, Gaithersburg, Maryland; AstraZeneca, Gaithersburg, Maryland; AstraZeneca, Gaithersburg, Maryland; AstraZeneca, Gaithersburg, Maryland; AstraZeneca, Gaithersburg, Maryland; AstraZeneca, Gaithersburg, Maryland; AstraZeneca, Gaithersburg, Maryland; AstraZeneca, Gaithersburg, Maryland; University of Southampton and University Hospital Southampton NHS Foundation Trust , Southampton, England, United Kingdom; AstraZeneca, Gaithersburg, Maryland; AstraZeneca, Gaithersburg, Maryland

## Abstract

**Background:**

AZD7442 is a combination of two half-life extended monoclonal antibodies, tixagevimab and cilgavimab. A population pharmacokinetic (popPK) model for AZD7442 included interim data from phase 3 trials in pre-exposure prophylaxis (PreP; PROVENT), post-exposure prophylaxis (STORMCHASER), and treatment of mild-to-moderate COVID-19 (TACKLE) in adults. This popPK model facilitated inclusion of adolescents in the AZD7442 label, the decision to increase the recommended PreP dose of AZD7442 in response to evolving SARS-CoV-2 variants, and selection of doses for a pediatric study (TRUST). Upon completion of the adult phase 3 studies, the popPK model was updated to include data from eight studies.

**Figure:**
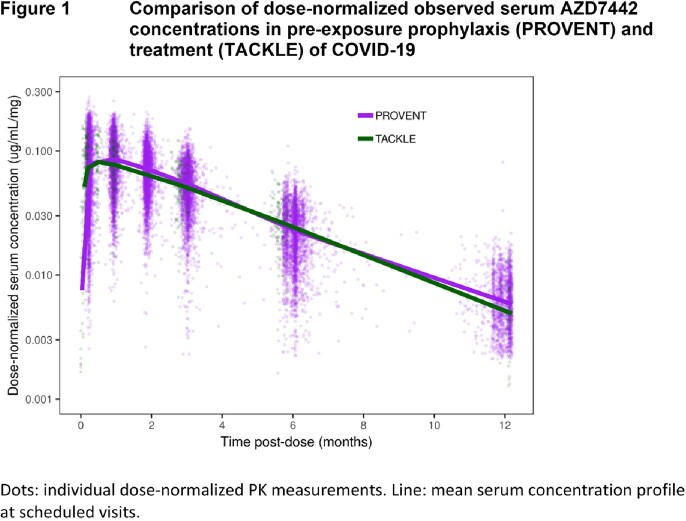

**Figure:**
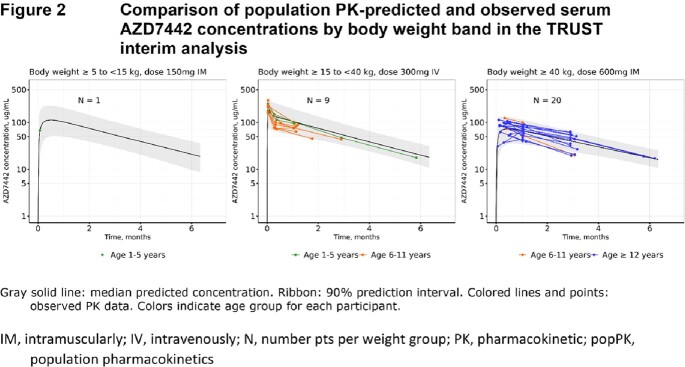

**Methods:**

The final popPK analysis included 4940 adult participants from North and South America, Europe, and Asia. To predict the PK of AZD7442 in pediatric patients, the effect of post-menstrual age on clearance was incorporated into the model.

**Results:**

The PK of AZD7442, tixagevimab, and cilgavimab were very similar and dose-proportional. PopPK analyses found no difference in AZD7442 PK between PreP and treatment (Figure 1). The final AZD7442, tixagevimab, and cilgavimab models included body weight, sex, age, BMI, Black race, and site of intramuscular administration as covariates; none of these were clinically relevant nor required dose adjustment. When interim TRUST (pediatric) data became available, popPK predictions were compared to observed serum PK data (Figure 2). Good agreement was observed, confirming appropriateness of dosing adolescents ≥40kg with the same dosing regimen as adults, and adequate characterization of AZD7442 PK in pediatric participants. Safety data from TRUST and adult studies were comparable.

**Conclusion:**

The PK of AZD7442, cilgavimab, and tixagevimab are comparable and follow linear kinetics with an extended half-life, allowing for prolonged duration of protection. AZD7442 PK is comparable across indications and between adults and pediatric populations. PopPK analyses facilitated rapid decision-making during the COVID-19 pandemic, including accelerating access to AZD7442 by adolescent patients ahead of the availability of pediatric clinical data.

**Disclosures:**

**Lindsay E. Clegg, PhD**, AstraZeneca: Employee|AstraZeneca: Stocks/Bonds **Oleg Stepanov, MS**, AstraZeneca: Employee|AstraZeneca: Stocks/Bonds **Henning Schmidt, PhD**, AstraZeneca: Employed by IntiQuan, which received payment from AstraZeneca for work involved in this analysis **Ventakesh Pilla Reddy, PhD**, AstraZeneca: Employee|AstraZeneca: Stocks/Bonds **Weifeng Tang, MD, PhD**, AstraZeneca: Employee|AstraZeneca: Stocks/Bonds **Michael Gibbs, PhD**, AstraZeneca: Employee|AstraZeneca: Stocks/Bonds **Sangeeta Sedani, MSc**, AstraZeneca: Employee|AstraZeneca: Stocks/Bonds **Antonella Nadia Tuillio, MD**, AstraZeneca: Employee|AstraZeneca: Stocks/Bonds **Sam Sadow, MA**, AstraZeneca: Employee|AstraZeneca: Stocks/Bonds **Nuria Martinez-Alier, PhD**, AstraZeneca: Employee|AstraZeneca: Employement|AstraZeneca: Stocks/Bonds|AstraZeneca: Stocks/Bonds **Taylor Cohen, PhD**, AstraZeneca: Employement|AstraZeneca: Stocks/Bonds **Saul N. Faust, FRCPCH PhD**, AstraZeneca, Janssen, Pfizer, Moderna, GlaxoSmithKline, Novavax, Sanofi, Seqirus, Medimmune, Merck, MSD, Iliad and Valneva: Advisor/Consultant|AstraZeneca, Janssen, Pfizer, Moderna, GlaxoSmithKline, Novavax, Sanofi, Seqirus, Medimmune, Merck, MSD, Iliad and Valneva: Investigator **Mark T. Esser, PhD**, AstraZeneca: Employee|AstraZeneca: Stocks/Bonds **Mats Någård, PhD**, AstraZeneca: Employee|AstraZeneca: Stocks/Bonds

